# Bio-waste to environmental purifier: Application of potato peel for acid red 73 adsorption from leather dyeing effluent

**DOI:** 10.1016/j.wri.2025.100281

**Published:** 2025-06

**Authors:** Fatema-Tuj Zohra, Sobur Ahmed, Md Zahangir Alam, Md Nurnabi, Nazia Rahman

**Affiliations:** a Institute of Leather Engineering and Technology, University of Dhaka, Bangladesh; b Department of Applied Chemistry and Chemical Engineering, University of Dhaka, Bangladesh; c Institute of Nuclear Science and Technology, Bangladesh Atomic Energy Commission, Dhaka, 3787, Bangladesh

**Keywords:** Adsorption capacity, Biosorbent, Dyeing effluents, Isotherm, Regeneration

## Abstract

Potato peel powder (PP) was prepared from kitchen wastes and explored in removal of Acid Red 73 (AR 73) dye from leather dyeing effluent. PP was characterized through FTIR, BET, SEM, XRD and EDX analysis. SEM micrographs illustrated rough and porous structure of PP that support the adsorption process. FTIR spectrum exhibited the changes caused by formation of hydrogen bonding, complex bonding, or other electrostatic interaction after dye adsorption. Wide and frail peaks in the XRD image indicated existence of amorphous carbon. BET analysis exhibited mesoporous structure of PP with average pore diameter of 85.98 Å. The impact of various influences viz. pH, dosages, concentration, time and temperature were explored through batch investigation. The maximum AR73 dye adsorption capacity of PP was 258.39 mg/g. The dye adsorption on PP complied both the Langmuir (R^2^ = 0.989) and Freundlich (R^2^ = 0.993) isotherm, preferably Freundlich model. The pseudo-second-order kinetic model (R^2^ = 0.999) was fitted better, i.e. chemisorption. The equilibrium adsorption capacity was 135.34 ± 1.59 mg/g at 298 K, which was decreased to 125.34 ± 1.81, 114.27 ± 1.43, and 104.08 ± 1.53 mg/g at 308, 318 and 328 K, respectively. Negative Gibbs free energy (ΔG) revealed that adsorption of AR 73 dye on PP was spontaneous. In real sample analysis, about 98.17 ± 0.58 % dye removal was obtained with 137.39 ± 2.46 mg/g capacity. This research revealed that PP has a good prospect for the application of AR 73 dye removal from leather dyeing effluent.

## 1 Introduction

The rapid industrialization, population growth, contaminated water resources, and climate change are interconnected and appeared as one of the most urgent issues in recent time. Globally, water depletion is increased significantly due to rapid growth of civilization and modernization. Therefore, a huge wastewater is discharged to the environment with several pollutants, such as dyes, heavy metals, hydrocarbons, phenols, detergents and other impurities [1,2]. Wastewater from leather, textile, plastic, paper, cosmetic, and printing industry are the sources of these pollutants [3]. Currently around 100,000 categories of dyes are commercially manufactured and 1.6 million tons of dyes are spent yearly, among them 10–15 % are released into water bodies after use [4,5]. Dye polluted effluents releases a huge suspended solids (SS), BOD_5_ and COD that decreases DO and increases turbidity of external water, which protects daylight penetration and reduces photosynthesis [6]. The abundance of dyes in effluents raises risk of bioaccumulation, desecrate aquatic system and jeopardizes the entire habitation [7,8]. Dyes could be classified as soluble dyes viz. acid, basic, direct, mordent and reactive dyes whereas azo, disperse, sulfur and vat dyes are the insoluble dyes [9]. The complicated chemical structure of dyes makes them mostly carcinogenic, mutagenic, stable and non-biodegradable, with negative environmental effects [10]. Due to its detrimental effects on various living species, dyes in effluents is a major problem, which need to be solved before discharging into the environment [11].

Research has concentrated on efficient ways to remove dyes from wastewaters by using a variety of technologies, such as ion exchange [12,13], membrane filtration [14,15], coagulation and precipitation [16,17], photocatalysis [18,19], oxidation [20], electrochemical oxidation [21], microbial or enzymatic degradation [22,23], and adsorption [24,25]. Adsorption is one of the most prevailing and expedient treatment processes for abatement of dyes from coloured wastewater [26]. To adsorb dye, heavy metals and additional filths from effluents, waste biomass such as peanut shell [27], cashew NUT shell [28], olive mill solid residue [29], Mangosteen peel waste [30], brewery wastes [31], kiwi branch biochar [32], acrylic acid grafted saw dust [33], jackfruit leaves [34], pine bark, oak ash and mussel shell [35], walnut shells [36], Rice husk biochar [37], onion peel and jute stick [38], banana peel reduced graphene oxide [39], and coconut fibre [40] have been utilized. Lignocellulosic biomass, such as waste from food production, agriculture, wood, paper, and cardboard made up to 15.7 % of all wastes produced in 2020 in Europe and it has a great deal of potential for use as a secondary raw material in industrial operations [41]. The potato is well known vegetables, which is cultivated more than 100 countries. Depending on the method employed to peel the potatoes, waste from peeling and trimming can vary from 15 % to 40 % [42]. Utilization of these wastes could be useful and effective, such as by preventing their accumulation in the surroundings and minimize the pollution resulting from them, and by using natural and affordable materials for diversified business [43]. Potato peel has been extensively studied as an fruitful adsorbent for various pollutants removal like, anionic congo dye [44], cationic dye Methylene blue and anionic dye Orange G [45], heavy metal Cd(II) [46], anionic dye Cibacron Blue P3R [47], Cu(II) ions [48], Pb(II) ion [49], methyl red (MR) dye [50], 4-nitrophenol [51], sorption of direct red-81 dye [52], diclofenac removal [53] from wastewater and so on. Moreover, as far as we are aware, none of these approaches on leather dyeing effluents for the remediation of AR 73 dye with bio-adsorbent PP.

In this research, potato peel powder (PP) was used for adsorption of acid red 73 (leather dye) from tannery waste water as leather processing industry mostly use acid dye in dyeing operation [54]. Adsorption ability of PP was appraised by different parameters for example pH, dosage, time, concentrations and temperature. The adsorption rate was evaluated with pseudo-first-order, pseudo-second-order kinetics equation and the thermodynamic parameters of free energy (Δ*G*), enthalpy (Δ*H*) and entropy (Δ*S*) are calculated at different temperature.

## 2 Experimental

### 2.1 Materials

A native tannery of Tannery Industrial Estate Dhaka (TIED), Savar, Dhaka provided the C.I. Acid Red 73 (AR73) dye. H_2_SO_4_ (98 %) and NaOH were bought from Merck, Germany. Potato peel was collected from house kitchen.

### 2.2 Preparation of adsorbent

Collected potato peels were cleaned and cut into small pieces. The pieces potato peels were oven dried at 60 °C for 48 h. Then the dried peels were powdered in stainless grinding machine and preserved in an air tight bottle. Powdered potato peels were employed as adsorbent to eradicate dye from aqueous solution and tannery effluents. A schematic representation of adsorbent preparation is shown in Scheme 1.

**Schime.1:**
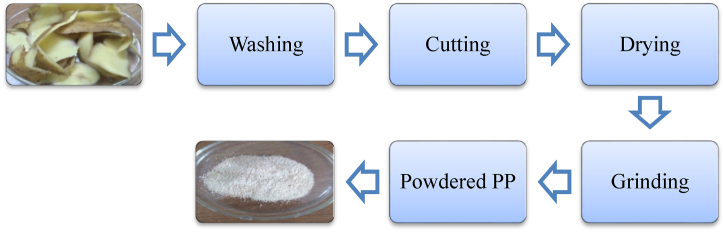
Schematic representation of PP adsorbent preparation.

### 2.3 Adsorbent characterization

Adsorbent PP were investigated through Fourier transformed infrared spectroscopy (FTIR) (8400S Shimadzu, Japan) to diagnose the existence of functional groups, scanning electron microscope (SEM) (JSM-6490LA, JEOL, USA) was utilized to recognize surface morphology, Surface area, pore volume, and pore size distribution of PP were investigated with BET sorptometer (model no. BET-201-A, PMI, USA). Elements composition of adsorbent was investigated by energy dispersive x-ray (EDX) (JEOL-JED-2300). X-ray diffraction of PP was executed on multipurpose X-ray diffraction system (Ultima IV) at the range of 05-100° to signify the crystallinity of the adsorbents. Zeta potential value of PP was assessed by Malvern Zetasizer Nano-ZS analyzer. UV–VIS spectrophotometer, (SHIMADZU, Japan) was deployed to measure dye concentration before and after adsorption.

### 2.4 Adsorbate solutions preparation

AR73 dye solutions of various concentration (100, 150, 200, and 300 mg/L) were equipped by diluting 1000 mg/L stock solution by mixing distilled water. Solution's pH was regulated by adding H_2_SO_4_ or NaOH with the assistance of HANNA pH meter (model-HI 98107). The physicochemical properties of Acid red 73 dye is mentioned in Table 1.

**Table 1:** Physicochemical properties of Acid red 73 dye.

Parameter	Properties
Chemical structure	
Molecular formula	C_22_H_14_N_4_Na_2_O_7_S_2_
Relative molecular weight	556.48 g/mol
Colour index	27290
Colour	Orange to Amber to Dark red

### 2.5 Batch adsorption

Batch adsorption was studied in a set of conical flasks with dye solution (20 mL). A definite amount of PP was poured to each flask and agitated for 2 h at 150 rpm. To optimize the adsorption conditions, each of the influencing factors of adsorption process; pH (2.0–7.0), adsorbent dosage (0.5–3.0 g/L), dye concentration (100, 150, 200, and 300 mg/L), contact duration (5–120 min), and temperature (298, 308, 318 and 328 K) were studied. The adsorption experiment process were replicated thrice at each phase. Whatman filter paper was utilized for filtration process and filtrate were analyzed by UV–VIS Spectrophotometer to measure unknown concentration of dye. After optimization of adsorbent PP, leather dyeing effluents also studied for dye removal from real sample. Dye adsorption efficiency and % of removal was deliberate through subsequent equations-

("Eq" .1) $$q_{t}=\frac{\left(C_{0}-C_{f}\right)\times V}{w}$$

where, q_t_ = adsorption capacity (mg/g), C_0_ = initial concentration (mg/L), C_f_ = final concentration of dye (mg/L), V = volume (L) of dye solution and W = mass (g) of adsorbent.

("Eq" .2) $$q_{e}=\frac{\left(C_{0}-C_{e}\right)\times V}{w}$$

where, q_e_ = adsorption capacity (mg/g) at equilibrium, C_e_ = concentration of dye (mg/L) at equilibrium.

("Eq" .3) $$\%\;of\;removal=\frac{\left(C_{0}-C_{f}\right)\times 100}{C_{0}}$$

## 3 Results and discussion

### 3.1 Characterization of potato powder peel (PP)

#### 3.1.1 Chemical composition

The principal component of potato peel is carbohydrate and protein. A good number of research proved that the composition of potato peel comprised of moisture, carbohydrate, protein, fibre, and fat are 11.20, 64.47–66.74, 12.50–13.52, 8.71, and 2.20 %, respectively [55,56].

#### 3.1.2 FTIR analysis

**Fig. 1:**
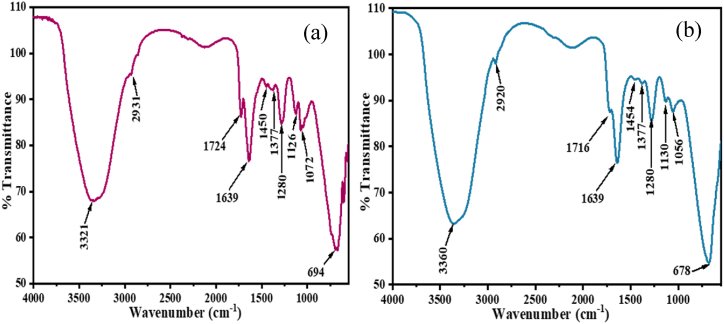
FTIR spectra of PP (a) before and (b) after dye adsorption.

FTIR spectrum is displayed many peaks reflecting the complex nature of PP in the range of 4000 to 600 cm^−1^ (Fig. 1). The peak at 3321 cm^−1^ in Figs. 1(a) and 3360 cm^−1^ in Fig. 1(b), was due to phenolic -OH stretching vibration which refers the existence of alcohols and phenols [57]. Band at 2931 and 2920 cm^−1^ were attributed saturated aliphatic -CH groups. The peak associated to -OH and -COOH were detected at 1639, 1450, 1280 and 1072 cm^−1^ in case of PP before dye adsorption, whereas, another significant peak was 1377 cm^−1^ of aliphatic C-H [58]. The FTIR spectrum of PP after dye adsorption exhibit changes due to may be complex bonding, such as H bonding and or other electrostatic interaction. The intensity of the peaks after dye adsorption were comparable to before dye adsorption.

#### 3.1.3 BET analysis

**Fig. 2:**
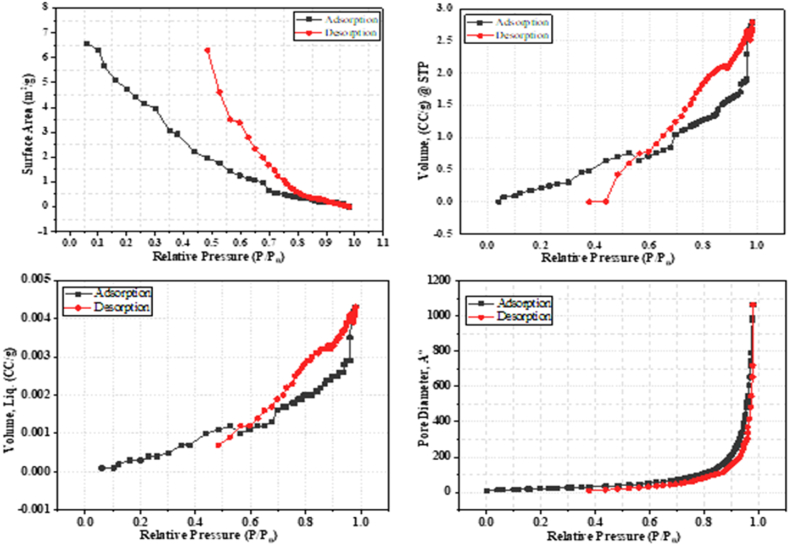
BET analysis.

The surface area, pore volume, and pore diameter of PP were examined by nitrogen gas sorption are presented in Fig. 2. The powdered PP had low surface area of 2.00 m^2^/g due to the breakdown of cell walls [59]. The average pore diameter of PP was measured 85.98 Å through Barrett-Joiner-Halenda (BJH) technique, which indicated PP as mesoporous [60]. The total pore volume (cc/g) and skeletal density of PP were 0.0043 cc/g and 1.8649 g/cc, respectively.

#### 3.1.4 SEM analysis

**Fig. 3:**
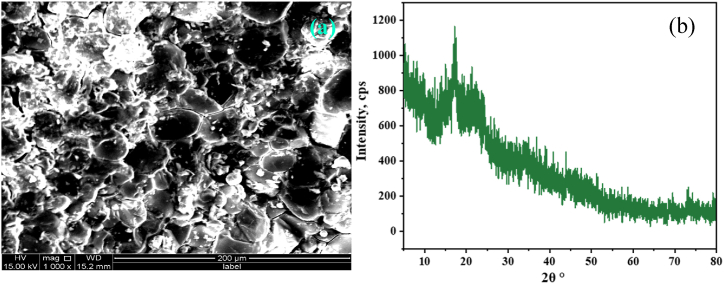
(a) SEM image 1000x, and (b) XRD analysis of PP.

The morphology of PP was captured through the SEM image (Fig. 3(a)) at 1000X magnification. The image showed a rough structure with numerous pores, which was supportive for the adsorption of dye on PP [48].

#### 3.1.5 X-ray diffraction (XRD)

There was no distinct peak associated with the crystalline segment in the XRD image. A bump within the dimension of 2θ° = 15–25° and a peak at 2θ° = 17.16° were detected (Fig. 3(b)) because of amorphous arrangement of PP. Wide and frail peaks in the XRD graph of PP suggest existence of amorphous carbon [61].

#### 3.1.6 Elemental analysis

**Fig. 4:**
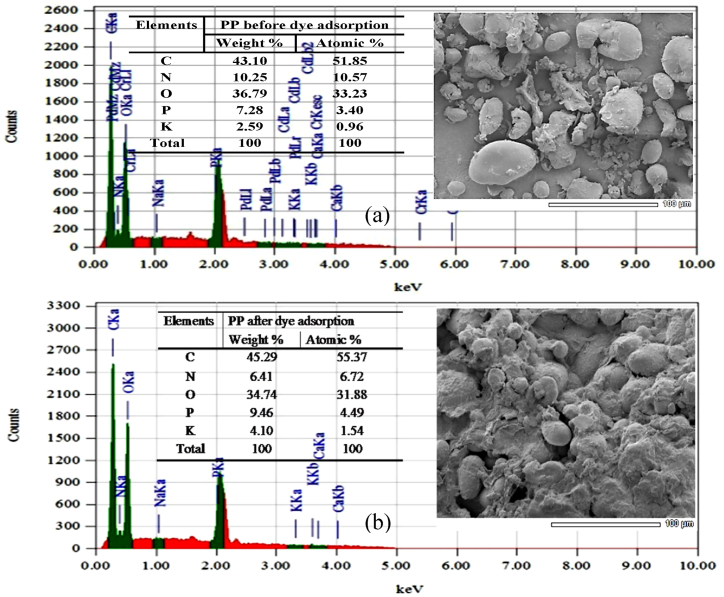
EDX spectrum of PP (a) before and (b) after dye adsorption.

It is noteworthy that EDX analysis assists to provide information about the composition of the adsorbent PP. The results demonstrated the presence of C, O, N, P and K, which are the main components of potato peels [62]. Dye laden EDX image of PP (Fig. 4) revealed the elemental composition after adsorption. Functional group of PP and the reactive groups of AR73 dye was responsible for the change in configuration of dye absorbed PP. There was a noticeable mass transfer of the C-group of dye and oxygen onto PP surface [63].

### 3.2 Batch adsorption

#### 3.2.1 Zeta potential value of PP

**Fig. 5:**
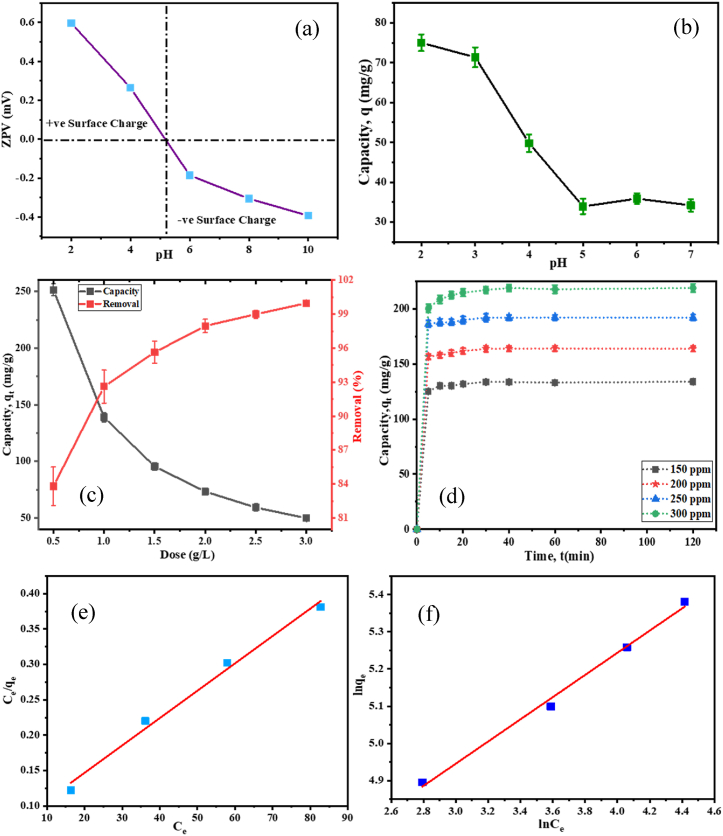
(a) ZPV of PP at different pH; Impact of (b) pH, (c) PP dosage, and (d) dye concentration and time on adsorption capacity, and (e) Langmuir, and (e) Freundlich isotherm for dye (AR73) adsorption on PP.

The role of pH on Zeta potential value (ZPV) of PP was studied. The study was performed at pH 2.0–10.0 through dispersing PP in deionized water (Fig. 5(a)). It was revealed that ZPV of PP was positive (0.264 mV) at 4.0 pH, whereas it was negative (−0.186 to −0.393 mV) at pH from 6.0 to 10.0. The pH of zeta potential value of pp was 5.2, where the net charge is zero.

#### 3.2.2 Effect of pH

The adsorbent surface and the nature of adsorbate have been influenced by pH [64]. To explore the effect of pH on PP, dye solution (150 ppm, 25 mL) was poured in the six conical flasks and pH controlled to 2.0, 3.0, 4.0, 5.0, 6.0, and 7.0 (Fig. 5(b)). After addition of PP (50 mg) in each solution, the mixture was shaken for 2 h at 160 rpm at room temperature and filtered with Whatman filter paper. The dye concentrations were determined by UV–Vis spectroscopy. The adsorption capacity of PP was decreased with increasing the pH (2.0–7.0) and the highest capacity was measured 74.99 ± 2.07 mg/g at pH 2.0. The principle approach to adsorb dye from solution was ion-exchange and H-bonding [65].

**Figure:**
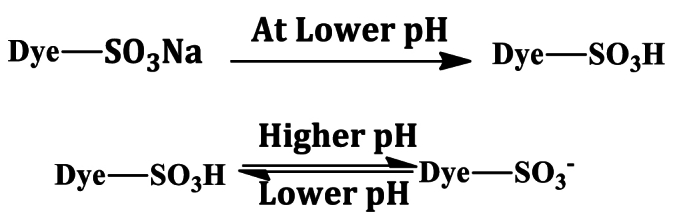

At lower pH (2.0), the adsorption capacity of PP was high in presence of greater number of protons. However, at higher pH surface negativity of PP was enhanced and anionic dye AR73 adsorption was decreased [66]. The adsorption could be increased at pH even lower than 2.0. However, this pH was selected as optimum to avoid extreme acidic condition. In this study, all batch investigations were executed at pH 2.0 to attain the maximum efficiency.

#### 3.2.3 Effects of adsorbent dosage

To analyze the effect of PP dosage on AR73 dye adsorption, different dosages (0.5–3.0 g/L) were applied and optimized the dosage. Batch trials were run for 2 h with 150 mg/L concentration at optimum pH 2.0. In Fig. 5(c) it is shown that the % of dye removal was increased from 83.81 ± 1.69 to 99.96 ± .02 %, whereas adsorption capacity was decreased 251.44 ± 5.32 to 49.98 ± 2.52 mg/g with the dosages increased from 0.5 to 3.0 g/L.

The availability of free adsorption sites contribute superior % of removal, however more unsaturated sites minimize the adsorption capacity of adsorbent [67]. It was revealed that almost 1.00 g/L dosage exhibits the best % removal and adsorption capacity. Therefore, 1.0 g/L dosage were selected as optimum and used in all over the study.

#### 3.2.4 Effect of concentrations and time

To evaluate the effect of dye (AR73) concentrations and contact duration on adsorption efficacy of PP, 20 mL solutions of four concentrations (100, 150, 200, and 300 mg/L) were taken in conical flasks and 20 mg (1.0 g/L) PP was poured to of each solution. The experiments were conducted at pH 2.0 at several time intervals ranging from 5 to 120 min with constant shaking speed.

According to the results (Fig. 5(d)), adsorption capacity was increased over time until it reached at equilibrium (after 30 min) and after which, no significant increment was found. The adsorption capacity was found to increase with increasing dye concentration. In case of 150 mg/L concentration, equilibrium adsorption capacity was 133.69 ± 1.59 mg/g, whereas it was 163.85 ± 2.74, 192.08 ± 3.46, and 217.26 ± 3.08 mg/g at 200, 250, and 300 mg/L concentration, accordingly. The maximum adsorption capacity was attained 217.26 ± 3.08 mg/g with 300 mg/L concentration.

#### 3.2.5 Isotherms

Adsorption isotherm expresses about correlation within the amount of adsorbates adsorbed on adsorbent, commonly examined the experimental data with Langmuir and Freundlich isotherm. The adsorbate is expected to be absorbed in monolayer without intermolecular exchanges in Langmuir, however, Freundlich isotherm reflects multilayer adsorption with irregular distribution [68]. Following equations were applied to analyze the isotherm of AR73 dye adsorption [69] -

("Eq" .4) $$\frac{C_{e}}{q_{e}}=\frac{1}{q_{m}b}+\frac{1}{q_{m}}C_{e\;}$$

("Eq" .5) $$R_{L}=\frac{1}{1+C_{m}b}$$

("Eq" .6) $$\ln\;q_{e}=\ln\;k_{F}+\frac{1}{n}\ln\;C_{e}$$

where, q_m_ = maximum adsorption capacity, C_m_ = maximum initial adsorbate concentration, k_F_ (L/g), b = Langmuir constant (L/mg), and n are the Freundlich constants.

Langmuir isotherm model was applied by Eq. (4), where the maximum capacity, q_m_ was calculated by plotting the value of C_e_/q_e_ vs C_e_ (Fig. 5(e)). Theoretical q_m_ was calculated from the slope and attained 258.39 mg/g with acceptable regression factor (R^2^ = 0.989). The separation factor R_L_ provides information of qualitative measure of the favorability, where R_L_ > 1 indicates unfavorable, and 0< R_L_ < 1 indicates satisfactory adsorption process. In this study, R_L_ was determined with Eq. (5) and the value was 0.0565, which illustrates satisfactory monolayer adsorption [70].

The experimental data was justified for multilayer adsorption through Freundlich isotherm Eq. (6) by a graph plotting with the value of lnC_e_ against lnq_e_ (Fig. 5(f)) and linear connection was observed (R^2^ = 0.993). From the slope, n was calculated as 3.359, which revealed good adsorption process [71]. Adsorption turn into further complicated as the value of n decreases, (n = 2–10 denotes worthy, n = 1–2 difficult and n < 1 poor adsorption) [72].

The value of Langmuir isotherm constants such as q_m_, b, R^2^ and R_L_ were 258.39 mg/g, 0.0556 Lmg^−1^, 0.989 and 0.0565, respectively, whereas Freundlich isotherm constant kF, n and R^2^ were 57.546, 3.359 and 0.993, respectively. Considering the values of different parameters of isotherm models, both the Langmuir and Freundlich models are followed for AR73 dye adsorption, preferably the Freundlich model.

#### 3.2.6 Adsorption kinetics for dye adsorption on PP

The adsorption kinetics is important to investigate how quickly ions move from aqueous to solid phase and how long it takes time to gain equilibrium position. Pseudo-first-order (PFO) and Pseudo-second-order (PSO) were implemented to justify the adsorption process. By means of Eq. (7), the PFO model was investigated [73]. The data obtained by applying equation (8) to the PSO adsorption kinetics model [74] was then verified. The linear appearance of PFO and PSO rate equations are-

("Eq" .7) $$\log\left(q_{e}-q_{t}\right)=\log\;q_{e}-\frac{k_{1}}{2.303}t$$

("Eq" .8) $$\frac{t}{q_{t}}=\frac{1}{k_{2}{q_{e}}^{2}}+\frac{1}{q_{e}}t$$

Here, k_1_ = rate constant of pseudo-first-order kinetics (L/min) and k_2_ = rate constant of Pseudo-Second-Order adsorption (g/mg min).

PFO kinetic Eq. (7) was introduced by Lagergren in 1898 to illustrate the adsorption process. PFO kinetic model was obtained by plotting a graph, where a linear relationship was observed among log(q_e_-q_t_) and t. In 1999 Ho and Mckay established PSO rate reaction (Eq. (8)) and the model was obtained with a graph by plotting t/q_t_ versus t. Different parameters of kinetic models were computed from the slop and intercept of each linear plot are encapsulate in Table 2.

**Table 2:** PFO and PSO kinetics parameters of dye adsorption on PP.

Kinetics model	Parameters	150 ppm	200 ppm	250 ppm	300 ppm
**Pseudo-First-Order**	q_e_^a^ (mg g^−1^)	133.69	163.85	192.08	217.26
	k_1_(1/min)	0.0965	0.0815	0.0571	01234
	R^2^	0.900	0.904	0.835	0.998
	q_e_^b^ (mg g^−1^)	12.078	12.204	7.889	30.478
**Pseudo-Second-Order**	k_2_ (g/mg min)	0.016	0.0138	0.015	0.0081
	R^2^	0.999	0.999	0.999	0.999
	q_e_^b^ (mg g^−1^)	135.14	166.67	192.31	222.22

a Experimental.

b Theoretical.

Outcomes of two models suggest that the theoretical q_e_ values better harmonized (Table 2) with the investigational q_e_ values, in case of PSO model. It was also observed that correlation coefficient (R^2^) values of PSO kinetic model was better compare to PFO kinetic model. Feasibility of the model is dependent on the closeness of theoretical and experimental q_e_ value and the highness of regression coefficient (R^2^). Hence, it is comprehensible that Pseudo-Second-Order model was well fitted for dye adsorption on PP compared to earlier model.

#### 3.2.7 Thermodynamic analysis

To verify endothermic or exothermic nature of dye adsorption on PP was also exercised at different temperature. Experiments were run at four different temperature to outline thermodynamic factors, particularly, Gibbs free energy (ΔG), enthalpy (ΔH), and entropy (ΔS) by dint of Eq. (9), Eq. (10) and Eq. (11).

("Eq" .9) $$\Delta G=RTlnk_{d}$$

Here, k_d_ = distribution constant, R = universal gas constant (8.314J mol^−1^K^−1^) and T = absolute temperature (K). k_d_ was deliberated by means of subsequent equation-

("Eq" .10) $$k_{d}=\frac{q_{e}}{C_{e}}$$

Enthalpy ΔH and entropy change ΔS was contemplated via van't Hoff equation-

("Eq" .11) $$\ln\;k_{d}=\frac{-\Delta H}{RT}+\frac{\Delta S}{R}$$

**Fig. 6:**
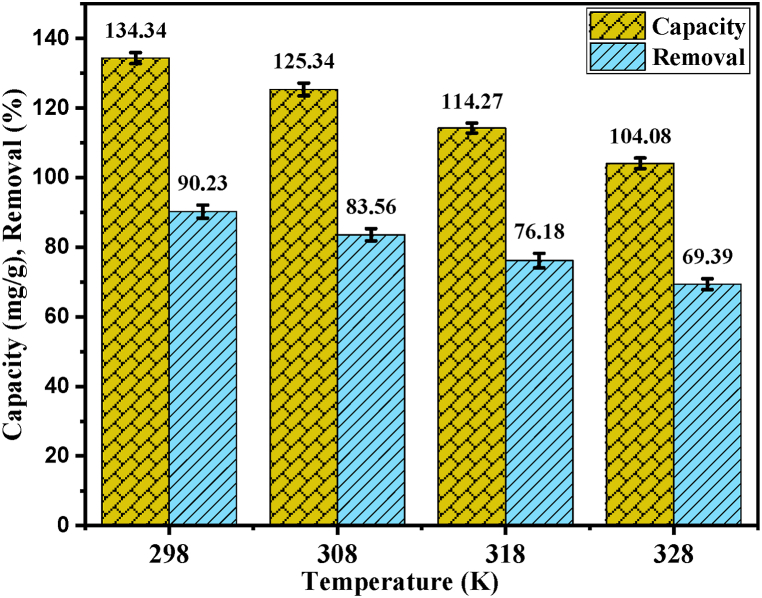
Percentage of removal and capacity of PP at different temperature.

For this study, 20 mg PP was added in to 20 mL (150 ppm) of dye solution at pH 2.0 for individual experiments and were shaken in a shaking incubator at 298, 308, 318 and 328 K temperature for 5–120 min. Impact of temperature and contact duration on dye adsorption capacity of PP were examined and found that the adsorption efficiency of PP was decreased with increasing temperature (Fig. 6). The kinetic energy increases and releases of the adsorbate from PP when temperature raises [71]. The equilibrium adsorption capacity was 135.34 ± 1.59 mg/g at 298 K, which was decreased to 125.34 ± 1.81, 14.27 ± 1.43 and 104.08 ± 1.53 mg/g at 308, 318 and 328 K, respectively. Similarly, % of removal of AR73 dye was 90.23 ± 1.89 % at 298 K, which was decreased to 83.56 ± 1.76, 76.18 ± 2.07 and 69.39 ± 1.58 % at the temperature 308, 318 and 328 K, respectively.

Standard enthalpy (ΔH) was calculated as - 29.498 kJ/mol from the slope and, the entropy (ΔS) was achieved from the intercept and measured −0.0815 kJ/mol. The results of ΔG for dye adsorption on PP at 293, 308, 323 and 338 K, were accomplished −5.503, −4.102, −3.074, and −2.231 kJ/mol, accordingly. Negative ΔG expressed that AR73 dye adsorption on PP was spontaneous and negative ΔH indicated exothermic process. Moreover, negative ΔS stated the lesser randomness of solid/solute interface during adsorption of dye on PP [75].

#### 3.2.8 Plausible mechanism

**Fig. 7:**
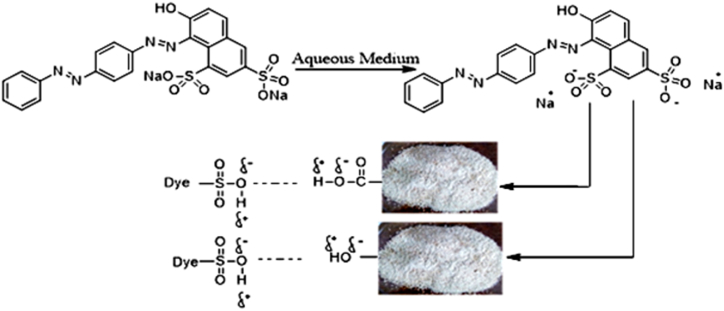
Plausible mechanisms of AR73 dye adsorption on PP.

The interaction among opposite charged particles via formation of various bonds, such as H-bonding, dipole-dipole attraction, ion-exchange, and Van der Waals forces are the causes of adsorption. The carboxyl, hydroxyl, and phenolic groups exist in bio-adsorbent can attach and remove dye along with other pollutants from dye effluents [76,77]. The hydroxyl and carboxyl groups of PP are responsible to form hydrogen bond (Fig. 7) with AR73 dye [45]. At lower pH surface charge of the PP developed positive charge, hence positive head of PP points towards bulk of the anionic dye solution. Therefore, electrostatic attraction between the negatively charged -SO_3_^-^ group of AR73 dye molecules and organic cationic groups on PP could be the favorable phenomenon for dye adsorption. Another mechanism could be the π–π interactions, where aromatic rings of phenolic compounds interact with the aromatic groups of dye through π–π interactions, which comprise charge transfer, dispersive force, and polar electrostatic components [78].

#### 3.2.9 Regeneration of used PP for dye adsorption

Regeneration of used adsorbents for further use is indispensable in developing any adsorbent commercially [79]. The scope of regeneration and reuse of PP was investigated by using 1.0 % NaOH and HCl solution. The adsorption capacity of regenerated adsorbent has been dropped steadily from 86.73 ± 2.61 to 37.49 ± 2.83 mg/g after 3rd cycle of reuse. Hence, the further study of regeneration of PP is required for improvement.

#### 3.2.10 Application of PP on real sample (dyeing effluents)

The performance of removing dye from real dyeing effluents was tested, after the assessment of AR73 dye removal capacity from solution. To observe the adsorption of dye from concentrated dye effluent, 250 mg (1.0 g/L) of PP was added to 250 mL of dye wastewater and followed by shaking for 2 h at pH 2.8.

The concentration of dye, TDS, EC, NaCl %, BOD_5_, and COD was investigated before and after adsorption (Table 3). The dye removal from dyeing effluents was 98.17 ± 0.58 % and the adsorption capacity was 137.39 ± 2.46 mg/g. However, this was much lower than theoretical capacity, q_m_ (258.39 mg/g) owing to the presence of aggressive ions and extra materials, which were used during leather processing [80]. Conversely, the water quality parameters such as total dissolved solids (TDS), electrical conductivity (EC), NaCl %, biochemical oxygen demand (BOD_5_), and chemical oxygen demand (COD) of treated dyeing effluents were also reduced immensely and controlled within the DoE standard limit (Table 4).

**Table 3:** Parameters of dyeing effluents before and after adsorption.

Parameters	Before adsorption	After adsorption	% of removal	DoE^a^ Standard
Dye (mg/L)	139.95 ± 2.46	1.61 ± 0.37	98.17 ± 0.58	–
Adsorption capacity (mg/g)	–	137.39 ± 0.74	–	–
TDS (ppm)	3854	48	98.75	2100
EC (μS/cm)	1709	97.3	94.31	1200
NaCl (%)	3.1	0.2	93.54	–
BOD_5_ (ppm)	812	37	95.44	≤100
COD (ppm)	2164	286	86.78	200–400

a Department of Environment.

**Table 4:** Comparison results of developed potato peels (PP) adsorbent with previous studies.

Adsorbent	Dye	pH	Maximum Adsorption capacity (mg/g)	Reference
Potato peel waste biomass	Acid Blue113	2.0	11.71	[81]
	Acid black1	3.0	1.79	
Potato peel waste	Methylene blue	8.0	33.55	[82]
HCHO treated potato peel (PP)	Methylene blue	8.0	47.62	[83]
H_2_SO_4_ treated potato peel (APP)	Methylene blue	12.0	41.60	
Potato peels (PP)	Methylene blue	6.0	179.9	[84]
	Orange G	2.0	40.5	
Potato peels (PP)	Cibacron Blue	2.2	100	[85]
H_3_PO_4_ activated Potato peels (PPa)			125	
Calcinated Potato peels (PPc)			270.3	
Potato peels (PP)	Acid Red 73	2.0	258.39	This study

## 4 Conclusions

In this investigation, PP was proved as a decent adsorbent to remove AR73 dye from aqueous solution and leather dyeing effluents. The maximum AR73 dye adsorption capacity of PP was 258.39 mg/g. The isotherm was exhibited the Freundlich behavior, which indicated a heterogeneous surface binding. The pseudo-second-order kinetic model was well fitted (R^2^ = 0.999), which demonstrated chemical coordination mechanism of sorption. Thermodynamically, it was found that the adsorption process was spontaneous, exothermic and viable. The used PP was regenerated and reused thrice. However, it could be reused with some fresh adsorbents. The performance of real dyeing effluents treatment with PP was very good. However, the study has some limitations such as low optimum pH and poor performance of regenerated adsorbent, which are need to resolved before the commercial application. Further studies are required for improving extra efficient, reusable and recyclable adsorbents to contribute in circular economy. Nonetheless, PP could be a useful adsorbent for treating dyeing effluents due to its simplicity and biodegradability.

## CRediT authorship contribution statement

**Fatema-Tuj Zohra:** Writing – original draft, Visualization, Software, Methodology, Investigation, Formal analysis, Conceptualization. **Sobur Ahmed:** Writing – review & editing, Writing – original draft, Validation, Methodology, Data curation. **Md Zahangir Alam:** Writing – review & editing, Visualization, Validation, Supervision, Project administration. **Md Nurnabi:** Writing – review & editing, Validation, Supervision, Methodology. **Nazia Rahman:** Writing – review & editing, Validation, Supervision.

## Declaration of competing interest

The authors declare that they have no known competing financial interests or personal relationships that could have appeared to influence the work reported in this paper.

## Data Availability

Data will be made available on request.
